# Oxytocin: Narrative Expert Review of Current Perspectives on the Relationship with Other Neurotransmitters and the Impact on the Main Psychiatric Disorders

**DOI:** 10.3390/medicina58070923

**Published:** 2022-07-11

**Authors:** Tudor Florea, Matei Palimariciuc, Ana Caterina Cristofor, Irina Dobrin, Roxana Chiriță, Magdalena Bîrsan, Romeo Petru Dobrin, Manuela Pădurariu

**Affiliations:** 1 Department of Medicine III, Faculty of Medicine, Grigore T. Popa University of Medicine and Pharmacy of Iasi, 16 Universității Street, 700115 Iasi, Romania; florea.tudor@umfiasi.ro (T.F.); matei.palimariciuc@umfiasi.ro (M.P.); caterina.cristofor@umfiasi.ro (A.C.C.); irina.dobrin@umfiasi.ro (I.D.); roxana.chirita@umfiasi.ro (R.C.); 2 Institute of Psychiatry “Socola”, 36 Bucium Street, 700282 Iasi, Romania; manuelapadurariu@yahoo.it; 3 Department of Drug Industry and Pharmaceutical Biotechnology, Faculty of Pharmacy, Grigore T. Popa University of Medicine and Pharmacy of Iaşi, 16 Universităţii Street, 700115 Iaşi, Romania; magdalena.birsan@umfiasi.ro

**Keywords:** oxytocin, receptors, serotonin, dopamine, review, glutamate, depression, behavior, stress

## Abstract

Is a cyclic neuropeptide produced primarily in the hypothalamus and plays an important neuromodulatory role for other neurotransmitter systems, with an impact on behavior, response to danger, stress, and complex social interactions, such as pair bonding and child care. This narrative expert review examines the literature on oxytocin as a brain hormone. We focused on oxytocin structure, distribution, genetics, and the oxytocin receptor system, as well as the relationship of oxytocin with other neurotransmitters and the resulting impacts on the main psychiatric disorders. Oxytocin levels have been correlated over time with mental illness, with numerous studies focusing on oxytocin and the pathophysiology of the main psychiatric disorders, such as autism, schizophrenia, personality disorders, mood, and eating disorders. We highlight the role oxytocin plays in improving symptoms such as anxiety, depression, and social behavior, as the literature suggests. Risk factors and causes for psychiatric disorders range from genetic to environmental and social factors. Oxytocin could impact the latter, being linked with other neurotransmitter systems that are responsible for integrating different situations during the development phases of individuals. Also, these systems have an important role in how the body responds to stressors or bonding with others, helping with the creation of social support groups that could speed up recovery in many situations. Oxytocin has the potential to become a key therapeutic agent for future treatment and prevention strategies concerning the main psychiatric disorders.

## 1. Introduction

Oxytocin is a hormone produced in the hypothalamus by the magnocellular nuclei and stored and secreted by the posterior pituitary, having a predominantly neuromodulatory role in the central nervous system and peripheral tissues. From a structural point of view, oxytocin is a polypeptide containing nine amino acids, which is synthesized as an inactive precursor (prooxyfizine). It consists of a longer sequence of amino acids, which are cleaved into oxytocin and neurophysine. The role of Neurophysine is to transport and store oxytocin in specific vesicles from where it is released in the systemic circulation when certain specific nerve impulses are triggered [1]. In response to various stimuli in the obstetrical sphere, such as breastfeeding, childbirth, lactation, and also non-obstetrical stimuli, including varying types of stress factors, oxytocin is released from pituitary into peripheral circulation, where it exerts its specific effects by binding to its receptors. It is important to note that there are other ways to release oxytocin centrally.

There is an intranuclear release of oxytocin but also a release by the dendrites and cell bodies of the supraoptic nucleus and the paraventricular nucleus. From this level, oxytocin reaches the hypothalamus, where it probably acts as an autoreceptor but also exists in other distant areas of the central nervous system, influencing a vast network of central oxytocin receptors [2]. The limbic regions and the brainstem also receive direct innervation of the oxytocin fibers. The phenomenon of local oxytocin release has also been identified in areas such as the amygdala, with particular relevance for, and association with, stressful situations [3]. In addition, other brain sites have been identified to produce oxytocin; among them, we mention the paraventricular nucleus with projections to other areas of the brain and also to the spinal cord and the nucleus accumbens, or the ventral tegmental area. Oxytocin is also produced in peripheral tissues where oxytocin receptors have also been identified. The areas involved in the local production of oxytocin are extremely diverse, including the pancreas and gastrointestinal tracts, and go far beyond the gynecological sphere, suggesting the multitude of biological processes in which oxytocin is involved compared to what was initially thought when it was first discovered [4].

Oxytocin is synthesized as an inactive neuropeptide. The process of activating oxytocin involves enzymatic cleavage into smaller fragments of the precursor oxytocin, and among these fragments is the oxytocin-carrying protein neurophysin 1, which is transported along with oxytocin in vesicles to their release locations. The final step in the enzymatic hydrolysis of the oxytocin precursor is mediated by the alpha-amidating monooxygenase peptidylglycine, which requires ascorbic acid as a cofactor [5]. Oxytocin is metabolized in the liver and plasma, with the help of oxytocinases and a much smaller percentage in the mammary glands. Oxytocin exerts its effects in the digestive system, in the reproductive organs (uterus, ovary, testis, placenta, amnion, corpus luteum, and mammary gland), where it is relevant in reproduction, in the cardiovascular system (heart and vascular endothelium), in renal, with a role in hydro-electrolytic regulation, and in muscular and adipose tissues, where it intervenes in metabolic processes [6,7].

Increased oxytocin levels in the central nervous system can be achieved pharmacologically with different routes of administration of oxytocin analogs or through behavior strategies such as music therapy, physical activities, gentle touching, massage, or interacting with pet dogs and cats [8,9].

## 2. Oxytocin and the Central Nervous System

Although endogenous oxytocin does not appear to easily cross the blood–brain barrier, circulating oxytocin may enter the spinal cord or stimulate the vagus nerve. Moreover, exogenous oxytocin may stimulate endogenous hypothalamic oxytocin secretion, but the mechanism by which this stimulation is achieved has not yet been clarified [10]. It can be speculated that exogenous oxytocin acts through oxytocin autoreceptors in the hypothalamic nuclei or indirectly via peripheral oxytocin receptors [11]. The central actions of oxytocin are varied and polymorphic; although there is considerable research trying to elucidate the impact of the oxytocin system, we are still far from fully understanding the clear roles and mechanisms of this system. In general, we can say that oxytocin modulates some neuroendocrine reflexes, and is involved in establishing complex social behaviors or coordinating behaviors related to reproduction, partner choice, childcare, or living in communities. For example, oxytocin receptors located in the paraventricular nucleus are involved in regulating the sexual responses of male and female rats and mice [12]. Oxytocin also exerts strong anti-stress effects, having modulatory effects on the hypothalamic-pituitary-adrenal axis [13,14].

The oxytocin system is involved in social behaviors by supporting affiliative behavior. Oxytocin is also important in group-related behaviors, selecting the attitude toward a stranger or group member. Exogenous oxytocin administration intensifies prosocial behavior, increasing it in a variety of species, including primates, rats, pigs, and sheep [15,16,17,18,19,20]. In humans, intranasal administration of oxytocin increases prosocial behaviors such as generosity, confidence, eyesight, and the ability to deduce the emotional states of others. Moreover, in humans, plasma oxytocin dosages have shown a link between oxytocin levels and parent–child relationships, romantic feelings of love and trust, empathy, and subsequent generosity towards strangers. These prosocial effects are not reported in all studies. However, some research has reported that oxytocin may induce adverse social reactions, especially when subjects have a hostile social environment [21]. It is clear that oxytocin has several physiological roles that have not been fully investigated and understood, and studies conducted in the field of psychiatry on various mental illnesses confirm this.

## 3. The Oxytocin Receptor

The oxytocin receptor is coupled to the IG class of proteins and has a proteic structure comprised of 388 amino acids with 7 transmembrane domains. The two ends of the oxytocin receptor are represented by the extracellular N-terminal domain, which has been described as relatively small in size, and the C-terminal intracellular domain, which is moderate in size, with its intracellular domain on the surface, on which phosphorylation sites important in enzymatic activation have been identified. The activated receptor is coupled with the IG protein and to the C-beta phospholipase and activates a secondary messenger system. The type of the secondary messenger is not yet known in the central nervous system, while in peripheral structures, there are several types of secondary messengers, including phosphatidylinositol [22]. The stimulation of oxytocin receptors that are functionally coupled to the G protein (Gq/11α) further determines the activity of the phospholipase C-isoform β. This activation chain further generates inositol trisphosphate and 1,2-diacylglycerol. Inositol trisphosphate triggers the release of calcium from intracellular deposits, while diacyl glycerol stimulates protein kinase C, which activates various proteins by phosphorylation.

Increased intracellular calcium levels may lead to the formation of calmodulin complexes that activate nitric oxide synthetase, both centrally and peripherally (e.g., in the endothelium). Nitrogen monoxide, in turn, stimulates guanylate cyclase to produce the cGMP complex. In the smooth muscle fiber, the calmodulin system triggers the activation of myosin, which will initiate the contraction of the smooth muscle [22]. These molecular processes described above also occur in myoepithelial cells within the myometrium or in the mammary gland, causing the ejection of milk. In the central nervous system, increased intracellular calcium levels are associated with increased cellular excitability and the promotion of gene transcription and protein synthesis. The gene encoding the human oxytocin receptor was first isolated and identified by Kimura et al., in 1992, using the cloning and expression strategy. The gene encoding the oxytocin receptor was located on the human chromosome 3p25-3p26.2 [23]. Descriptively, the oxytocin receptor gene contains three introns and four exons. Exons 1 and 2 correspond to the uncoded region, while exons 3 and 4 encode the amino acids of the oxytocin receptor, and exon 4 alone contains the coding sequence for the terminal portion of the seventh transmembrane domain as well as the entire uncoded region, including the polyadenylation signals. Intron 3 is the largest, measuring 12 kb, and is involved in cleavage as it encodes the region immediately after the transmembrane domain [15]. Subsequently, after the gene coding structures for the human oxytocin receptor were identified, the coding sequences for the oxytocin receptor in other animals followed, including pigs, rats, sheep, cattle, mice, and rhesus monkeys [15,16,17,18,19,20]. Looking at the results of the studies on the oxytocin receptor in several animal species, it appears that there are no significant differences in the structure of the oxytocin receptor between species. There are currently data that support the fact that, structurally, there is only one type of oxytocin receptor in all species, and the differences between species regarding the oxytocin receptor are only in the distribution pattern. Another important concept that distinguishes the oxytocin receptor between species is the differences in the regulation of the oxytocin receptor in different species, which causes various internal or external factors, such as hormones or neurotransmitters, or environmental factors to determine typical patterns of receptor distribution [24].

The number of receptors varies in the same individual at different stages in their life, having a key role in biological events related to reproductive function such as procreation, pregnancy, or lactation in the dynamics of the oxytocin system. These changes in the body lead to an increased expression of oxytocin receptors, both peripherally and in various areas of the brain. The condition for the high-affinity receptor requires both Mg2+ and cholesterol, which probably function as allosteric modulators. The function and physiological regulation of the oxytocin system are highly dependent on steroids. The binding region of the receptor agonist appears to be an area subject to mutagenesis and molecular modeling, and is different from the binding site of the antagonist [25,26,27].

Some genetic variants of the oxytocin system may be of particular importance in mental illness. Oxytocin receptor gene variation may modulate the signaling properties of oxytocin receptors. Thus, one of the variants of the oxytocin gene, *rs53576*, located in the third intron of the gene, is among the most studied variants of the oxytocin receptor gene, and it seems that it could indicate a phenotype predisposed to stress. The SNP variant *rs53576* has three types of alleles: GG, AG, and AA. Based on different studies, it has been hypothesized that the *G rs53576* allele, which is associated with oxytocin levels in humans, is beneficial in prosocial behavior, but in some studies, it has also been found to be associated with maladaptive behavior [28]. Although the mechanism by which oxytocin receptor variants influence different traits in subjects is still unclear, the GG genotype was associated with a decrease in oxytocin receptor DNA methylation, which offers increased resilience to environmental stress, especially during developmental stages in humans [29]. In different behavior studies, A allele carriers appear to have a higher sensitivity to stress when social support is provided, have fewer social skills, lower optimism, lower self-esteem, and at the same time have a higher risk for mental illness than people with the GG allele [28,30,31]. Other evidence supporting the association of the AA allele with vulnerability to stress also comes from Tost and his collaborators, who have shown that people who carry the A allele have a structural reduction in the volume of the hypothalamus and increased connectivity between the hypothalamus, the amygdala, and the dorsal part of the anterior cingulate cortex [28].

Oxytocin receptors have been identified using autoradiography techniques of the receptor and by their expression of mRNA in various areas of the brain. Some brain regions have been specifically identified as having a higher oxytocin receptor density, including olfactory bulbs, anterior olfactory nucleus, olfactory tubercles, nucleus acumbens, prelimbic cortex, ventral subiculum, the amygdala’s central nucleus, the ventromedial hypothalamic nucleus, cingulate cortex, the dorsal motor nucleus of the vagus nerve, and the solitary tract nucleus [32]. Studies show that the oxytocin receptor has a wide distribution in the brain, with expression in various areas of the brain, including neurons in the olfactory processing regions (olfactory nuclei and piriform cortex), in the limbic brain (including the amygdala, septum, and the preoptic medial nucleus), in the hippocampus and hypothalamus, in the brainstem (including the vestibular nerve, the motor and sensorial nuclei of the cranial nerves that regulate the sensorial and motor functions of the face and mouth, and the autonomic centers, such as the solitary tract nucleus). The highest density of oxytocin receptors is present in the ventromedial hypothalamus [33]. This pattern of distribution also suggests the special role that the oxytocin system plays in relating and adapting to the environment and the specific social context.

Oxytocin receptors are also extensively present in the peripheral tissues, in areas that are also involved in homeostasis regulation. Thus, oxytocin receptors are found in the ovary, testis, adrenal glands, uterus, mammary gland, liver, and fat cells but also in the circulatory system [20]. The roles associated with peripheral distribution are better known and easier to study compared to the relevance of the system in neuro-psychic processes, hence the importance of elucidating the neurobiological processes involving the oxytocin system. The major and versatile roles of the oxytocin system in the individual are also supported by the differences in its distribution in different mammal species. It also appears that there are differences between subspecies in the regulation of the oxytocin receptor, allowing for a variety of factors to determine typical patterns of distribution of these receptors. For example, in the case of prairie and mountain mice, the different distribution of oxytocin receptors is consistent with important differences in social affiliation behavior. The distribution of the oxytocin receptor was compared in two types of field mice and reconfirmed by comparing the distribution of oxytocin type receptors to two other subspecies of field mice. In prairie mice, the density of oxytocin receptors was highest in the prelimbic cortex, the nucleus of the terminal stria, the nucleus accumbens, the middle nucleus of the thalamus, and the lateral areas of the amygdala. These regions of the brain showed lower ligand binding in field mice, in which oxytocin receptors were located predominantly in the lateral septum, ventromedial nucleus of the hypothalamus, and in the cortical nucleus of the amygdala. These results indicate that the distribution of the oxytocin system is an important mechanism in the evolution of different species in adapting to social context and affiliation behavior [34].

In terms of ligand selectivity, the oxytocin receptor has a poor selectivity profile. From the whole structure of the receptor, what gives selectivity is the cyclic part of the receptor and the amino-terminal end. The amino-terminal domain and the first extracellular loop of the oxytocin receptor appear to interact with the carboxy-terminal linear tripeptide part of the oxytocin structure, while the second extracellular receptor loop appears to interact with the cyclic domain of oxytocin [35].

Oxytocin also appears to mediate the process of choosing a partner by gathering relevant social information. The choice of partners could also be influenced by past and present social experiences. The decisions of choosing the partner of an individual can be influenced by what is considered desirable by the social group, or even can be influenced by the mechanism of “copying”, where recognizing potential partners occurs by observing the choices made by another subject; the process is called “partner choice copy”. It seems that the neurobiological mechanisms underlying this decision-making process could focus on oxytocin, which processes relevant social information and favors certain decisions in choosing a partner [36]. The central actions of oxytocin range from modulating neuroendocrine reflexes to establishing complex social and bonding behaviors related to reproduction and childcare. Oxytocin also exerts strong anti-stress effects that can facilitate pair bonding. Overall, the modulation of sex hormones is one of the most important features of the oxytocin system. Oxytocin receptors located in the paraventricular nucleus are involved in regulating the male and female sexual responses. From all this data on the oxytocin system, it is obvious that oxytocin has several physiological roles that have not been fully investigated. The central oxytocinergic circuit connects areas of the hypothalamus (paraventricular nucleus) with the ventral tegmental area through dopaminergic neurons, forming the mesolimbic pathway. The activation of this mesolimbic pathway by oxytocin and by its interaction with dopaminergic nuclei causes oxytocin to have special implications for facilitating sexual and social behavior. The oxytocin receptor gene is differentially expressed in various tissues. For example, in the uterus or hypothalamus, the oxytocin receptor correlates with the level of sex steroids but is particularly correlated with estradiol. The oxytocin receptor gene is also modulated by interferon γ, as shown by an in vivo study that found that interferon γ suppresses the production of oxytocin receptor mRNA [37].

## 4. Connections between Oxytocin and Neurotransmitters in Affective Disorders

The notion that oxytocin is a mediator for other neurotransmitter systems and that its key role may be to integrate the effects of other molecules, has been speculated by many researchers who have investigated the complex link between the oxytocinergic system and various neurotransmitters in several experiments. The neuromodulatory role is speculated through oxytocin’s differentiated effects, including social, emotional, and behavioral effects, which differ greatly depending on the context of the experimental situation but also on various other factors. These affective carrying events have a certain relevance in activating the oxytocin system when they are labeled by the subject as having certain meanings. Thus, stimuli that can be interpreted by the subject as potentially dangerous, such as the presence of a stranger or a person with traits that suggest mistrust, or a hostile environment, will trigger a completely different oxytocin-mediated reaction compared to the presence of either a very close person or positive facial expressions that indicate a friendly environment. The fact that oxytocin can cause different reactions depending on how the environment is perceived by the subject is based on the hypothesis that oxytocin plays its role indirectly, orchestrating the activity of several neurotransmitter systems [38,39].

### 4.1. Oxytocin and the Glutamatergic System

Taken together, glutamate, oxytocin, and GABA (gamma-aminobutyric acid) are considered to be the most important neurotransmitters and neuromodulators in situations of stress, danger, or conflict, but they also have a role in new life events that impose adaptation. These molecules play an essential adaptive role, and disturbances in the functionality of this system can lead to negative consequences of functioning, especially in the situation where the body should be able to defend itself. In other words, their primary role is manifested especially when the harmful conditions exceed the body’s ability to adapt. When the body fails in the face of stressor, pathophysiological changes, such as inflammation, oxidative stress, tissue degeneration or damage, or cellular apoptosis, occur. These changes caused by exhaustion are mediated by glutamate, which is released in the prefrontal cortex. Under conditions of intense stress, very large amounts of glutamate are released, having toxic effects, especially on neurons, causing degeneration and neuronal death. Studies show that oxytocin may play a modulating role in stressful situations, emotionally modulating the individual’s perception of danger. Oxytocin can alleviate the feeling of fear in general but also has an adaptive role to stress, which could be related to the glutamatergic system. There also appears to be a pattern of oxytocin receptor distribution that indicates colocation with the glutamatergic receptors. Studies have shown that exposure to stress increases the expression of oxytocin in the suprachiasmatic and paraventricular nuclei of the hypothalamus [40,41,42]. It appears that, contrary to the cytotoxic effect of glutamate, oxytocin has neuroprotective effects by increasing neuronal resistance to various toxins. This has been demonstrated in studies involving fetal hypoxia [43]. The antagonizing effect of glutamate is also supported by the colocalization pattern of the two neurotransmitter systems. All these data support the fact that glutamate and oxytocin are of particular importance in the body’s reaction to psychostressing factors.

Glutamate receptors, such as N-methyl-D-Aspartate (NMDA), are known to be actively involved in learning and memory. How they are involved in the information retention process is dependent on protein kinase C, which is activated by the increased influx of Ca2+ from the extracellular level, with the subsequent triggering of a series of intracellular reactions that cause memory formation. In an in vitro experimental setting, the application of a relatively high concentration of oxytocin (>1 μM) reduces the influx of molecular calcium due to the stimulation of NMDA receptors via glutamate. In the same experiment, oxytocin also decreased the binding of a ligand ([3H] 4-beta-phorbol 12,13-dibutyrate) to NMDA receptors, which demonstrates the translocation of protein kinase C within the cell membrane. All these data suggest that oxytocin reduces the activity of NMDA receptors and may interfere with memory and learning processes [44].

A double-blind study testing oxytocin’s involvement in memory formation was conducted on 38 men who received either intranasal oxytocin (24 IU) or a placebo before incidental learning. The results obtained indicate that oxytocin significantly affected learning performance compared to the placebo and that this result is not influenced by the type of stimuli used (associated or not with the reproductive concept) [45]. Neuropeptides such as oxytocin or vasopressin act as neuromodulators on neurotransmitters such as glutamate or GABA, only at higher stimulation frequencies. Glutamatergic neurons in the paraventricular nucleus are also involved in the control of oxytocin release [46].

The results obtained by Yoshida and his collaborators suggest that there may be a disturbance of the oxytocin system in subjects with a depressive and anxious phenotype, but at the same time, a disturbance in the release of glutamate because of the administration of oxytocin agonists, does improve stress behavior only in pathological subjects and not in normal ones. The study’s authors speculate on the potential utility of oxytocin receptor agonists in the treatment of stress-related psychiatric disorders in adulthood [47].

### 4.2. Oxytocin and the HPA Axis

Oxytocin may play a modulating role in stress responses. Summarily at the biological level, oxytocin could modulate the reactivity of cortisol and cytokines, while at the social level, the oxytocin system would increase the level of confidence and empathy, which are generally described as prosocial behaviors. In a 2003 study by Heinrichs and colleagues, male subjects who were exposed to social stress, followed by social support and intranasal oxytocin, had a less pronounced increase in cortisol levels than men who received either social support or oxytocin alone or none of these treatments [48]. In another experimental setting, children exposed to a situation of social stress were tested. As a result, children who could see or hear their mothers’ voices had higher levels of oxytocin and lower levels of cortisol compared to children who had no contact with their mothers [21]. Oxytocin can be seen as having an anti-stress effect, attenuating stress-related responses in familiar environments, but can also amplify stress reactions, having the role of protection against potentially harmful stimuli in environments perceived as hostile. A relevant example in this regard is shown by Cardoso and his team, who showed that in the situation of increased salivary cortisol levels, this was due to the action of various stressors, for example, either physical stress or mental stress resulting from social ostracization, or conjugal conflict. In these conditions, intranasal oxytocin administration decreased the level of cortisol in all experimental situations, limited the increase in cortisol caused by social ostracism, and increased the concentration of cortisol after the conjugal conflict resolution [49].

### 4.3. Oxytocin and the Serotonin System

The serotonergic system has long been shown to be important in stress-related mental disorders such as depression and anxiety. The relationship between oxytocin and serotonin could explain the importance of oxytocin in affective disorders. Research in recent years on animals has shown the anatomical links between oxytocin and serotonin. The serotonergic fibers that leave the dorsal and medial raphe nuclei in the bulb reach the magnocellular neurons in the supraoptic and paraventricular nuclei in the hypothalamus, where oxytocin is released. In this region, serotonergic fibers overlap and follow the distribution of oxytocin fibers [50]. Jørgensen and colleagues demonstrated that serotonergic neurons in the raphe nuclei express oxytocin receptors, suggesting the importance of oxytocin in the release of serotonin [51]. Other studies have shown that the administration of oxytocin in the postnatal period has the effect of increasing the length of serotonergic axons in areas of the brain relevant for serotonergic and oxytocic transmission, namely the hypothalamus and amygdala [52]. These findings indicate that the anatomical substrates of oxytocin and serotonin present an anatomical and functional interface that explains the importance of these two systems in regulating emotional behaviors.

The mechanisms by which oxytocin may influence serotonergic transmission could be explained by either the direct release of serotonin through the action of oxytocin on serotonergic receptors, or indirect release by increasing the availability of 5HT1A receptors. This model of oxytocin–serotonin interaction could at least partly explain how oxytocin may have anxiolytic properties [53]. In the paraventricular nucleus of the hypothalamus, oxytocin cells have serotonergic receptors (5HT1A and 5HT2A), which suggests serotonin’s importance in the release of oxytocin from the hypothalamus. Serotonin, serotonin precursor, and serotonergic agonists stimulate oxytocin release, while serotonin antagonists inhibit oxytocin secretion. These effects are also valid for vasopressin [51]. Moreover, it seems that excess serotonin causes disorders of the oxytocin system [54].

There is a high degree of overlap between serotonin transporter (SERT) fibers and oxytocin cells in the paraventricular and supraoptic nuclei of the hypothalamus, as shown by modern immunocytochemical and immunofluorescence techniques. Evidence that supports the serotonin–oxytocin relationship come also from clinical trials involving antidepressants. Yoshida and colleagues have shown that selective serotonin reuptake inhibitors (SSRIs), such as citalopram and fluvoxamine, appear to exert some of their antidepressant effects by releasing oxytocin [47]. Another study supporting this hypothesis observed that citalopram administration resulted in a statistically significant increase in plasma oxytocin and cholecystokinin levels, without differences between the acute and chronic phases of treatment. This suggests that the release of oxytocin may be a mediator of the pharmacological actions of SSRIs and emphasizes once again the importance of the oxytocin system in affective disorders and the role that oxytocin may play in the treatment of these disorders [55]. In another study, parenteral administration of 5-HT increased the release of oxytocin during breastfeeding, whereas administration of the 5-HT receptor antagonist or the depletion of serotonin by the administration of p-chlorophenylalanine inhibited lactation [56]. These effects are thought to be mediated by the 5HT-1A and 2A receptors. Clinical and experimental animal studies have shown that the administration of specific agonists for these receptors resulted in dose-dependent elevated plasma oxytocin levels. Moreover, the effects of agonists were blocked by the prior administration of 5-HT1A and 5-HT2A/2C receptor antagonists [57].

Yoshida et al., showed that the anxiolytic action of oxytocin could be mediated by the 5-HT2A/2C receptors. A fluorescent protein called Venus cDNA replaced part of the gene encoding oxytocin receptors to determine the pattern of OXT-R expression throughout the brain in a mouse experimental model developed for this specific context. The authors observed widespread expression of oxytocin receptor-containing cells. More than half of the tryptophan hydroxylase-positive neurons, positive for the Venus protein, were found in the raphe nuclei. Intra raphe oxytocin infusion increased serotonin release in the median raphe nuclei. The administration of 5-HT2A and 2C receptor antagonists inhibited this effect. This has led the authors to speculate that oxytocin-induced increased serotonergic activity may underline its anxiolytic effects [47]. Dolen et al., studied the preference of mice for a particular place associated with social interaction rather than a room previously associated with social isolation. This task measures the rewarding effects of social interactions. The study found that the ablation of presynaptic oxytocin receptors in the projections from the dorsal raphe nucleus to the nucleus accumbens is sufficient to eliminate this preference. They performed additional experiments suggesting that oxytocin acts by increasing the release of serotonin from dorsal raphe nucleus projections to the nucleus accumbens and that serotonin acts on 5-HT1B receptors in the nucleus accumbens, causing long-term depression of excitatory synapses [58].

Another experiment performed on human subjects used positron emission tomography to compare the effects of exogenous oxytocin administration as a modulator on serotonergic neurotransmission in patients with autism spectrum disorder versus control patients. A modulatory effect of oxytocin on serotonin was observed only in the healthy subjects. Patients with autism showed no changes in serotoninergic transmission after oxytocin administration. These results once again support oxytocin’s modulatory action over serotonin and its involvement in autism spectrum disorders [59]. Similarly, rats given a serotonergic agonist (5-methoxytryptamine) show decreased oxytocin in the paraventricular nucleus of the hypothalamus, increased serotonin plasma levels, and autism-like behavior [60], suggesting bilateral interaction between oxytocin and serotonin. Male and female mice that have excess serotonin also have a lower number of oxytocin-producing cells in the paraventricular nucleus of the hypothalamus, but only females appear to have the ability to self-regulate serotonin receptors to excess systemic serotonin in a way that promotes oxytocin cell survival and functional efficiency [61].

The link between oxytocin and serotonin needs to be better investigated due to multiple implications in terms of psychiatric pathology, especially knowing serotonin’s importance in the etiopathogenesis of depression, anxiety, addictions, autism, aggression, and suicidal behavior. Deciphering the relationship between oxytocin and serotonin could advance our knowledge about the biological mechanisms in some mental disorders and, at the same time, could have a significant impact on expanding the therapeutic arsenal and even increasing the individualization of psychotropic treatment.

### 4.4. Oxytocin and Brain-Derived Neurotrophic Factor

Brain-derived neurotrophic factor (BDNF) is a member of the neurotrophin family of growth factors, found in the central nervous system, and supports the growth, survival, and differentiation of neurons, playing an important role in neuronal plasticity, which is essential for learning, memory, and higher thinking. BDNF binds tropomyosin receptor kinase B (TrkB), which has implications for the development of various neuropsychiatric and neurodegenerative disorders or other proliferative or epileptogenic conditions [62]. BDNF has been linked to the etiology of depression and anxiety, and recent studies proposed its use as a pharmacological agent or as a biomarker. [63] Its role as a biomarker was further investigated in the addictive cycle. Higher BDNF levels were found during the withdrawal phase, possibly predicting the risk of dangerous behaviors and relapse. In the case of abstinence, BDNF levels were observed to be inversely related to the severity of addiction [64]. Oxytocin also plays a role in neuromodulation in substance abuse, using the glutamate receptors, and is involved in memory forming and learning, indirectly opposing drug-induced behavior. More pre-clinical studies have evaluated the effects of oxytocin on central neuromodulation from transition states of methamphetamine dependence, alleviating intense withdrawal symptoms with promising results in rats [65].

BDNF was also associated with PTSD; patients with higher serum levels showed lesser symptoms with a higher degree of impulsiveness. It was proposed that increased impulsiveness may be a protective psychological mechanism for such cases and may represent a positive indicator of boldness, quickness, and spontaneity [66]. Moreover, recent evidence shows that BDNF, similar to oxytocin, regulates social and maternal behavior, eating behavior as well as obesity, impacting other psychiatric disorders such as autism and schizophrenia and, as mentioned, mood and anxiety disorder. A series of animal studies found that oxytocin influences the expression of BDNF in rat mothers, where oxytocin antagonists in the ventral hippocampus significantly reduced BDNF gene expression, which could have a significant impact on maternal behavior and resilience to the stress of reproduction [67]. On the other hand, TrkB signaling in the hypothalamic oxytocin neurons of female mice impacts gene expression at this level, influencing sex-specific social behaviors [68]. The BDNF–oxytocin link could have great potential for understanding the underlying mechanisms of various psychiatric disorders and should promote further investigation on the subject.

## 5. Oxytocin’s Relevance in Mental Disorders

### 5.1. Oxytocin and Autism

Over the last 20 years, there has been a lot of research on the link between oxytocin and autism. In 1998, Modahl tested the level of oxytocin in the blood of autistic children and observed significantly lower plasmatic oxytocin levels in autistic children compared to the control population [69]. Subsequently, in 2003, in another experimental setting, autistic repetitive behaviors decreased after oxytocin administration. This also had beneficial effects on adults with autism, but these effects manifested especially in the ability to effectively understand and interpret the voice tone [70].

These hypotheses and clinical data are also partially supported by functional neuroimaging. The effect of oxytocin on the mesocorticolimbic system was examined, especially under the conditions of rewards given to subjects suffering from childhood autism. Studies have shown a link between dopamine and oxytocin and the relevance of mesocorticolimbic brain regions in the potential mechanisms of action of oxytocin in autism spectrum disorders. During tasks of socio-emotional recognition in autistic patients, there was an increase in activation in brain areas such as the ventral striatum or left premotor cortex in response to intranasal oxytocin administration. The same regions of the brain showed a decrease in activation when the task is performed in non-social conditions, where patients only interacted with objects [71]. Also, intranasal administration of oxytocin to subjects with autism increased functional connectivity between the ventral striatum and the ventromedial prefrontal cortex, demonstrating the importance of the mesocorticolimbic regions of the brain in the mechanism of action of oxytocin. [72].

The social component of oxytocin’s action may have a major impact on autism, where social interaction plays an important role. Many studies have failed to identify the clinical benefits of oxytocin towards general social behavior in adults with autism [73]. However, a recent study found beneficial effects of oxytocin towards social functioning in autism, with these effects also being influenced by endogenous vasopressin levels [74]. The mechanisms of action of oxytocin in influencing social functioning are not fully understood. Some theories try to explain how oxytocin influences the dynamics of the social response. A potential mechanism of action may be the ability of oxytocin to influence sensitivity to external rewards and thus directly facilitating reward-based learning behavior. Preclinical studies suggest the mesocorticolimbic dopamine system as a mechanism by which oxytocin exerts its prosocial effects [75]. Mesocorticolimbic oxytocin and dopamine interact in such a way that activation of oxytocin neurons in the ventral tegmental area increases dopaminergic activity in the mesocorticolimbic system. Moreover, when an oxytocin receptor agonist is administered, mice show a subsequent decrease in dopaminergic release in the nucleus accumbens, suggesting oxytocin’s significance in the mesocorticolimbic transmission of dopamine [76].

Studies have shown that oxytocin does not immediately have a prosocial role but modulates the individual’s response to various social stimuli related to the conservation instinct, both in human and animal models. Due to the need for effective treatments for the symptoms of autism spectrum disorders, there has been a growing interest in the potential of oxytocin to ameliorate the deficiencies in social communication. To summarize, studies of autism and the implications of oxytocin have shown beneficial effects on certain components of social functioning when oxytocin is administered. Benefits in social functioning, including increased emotional recognition or confidence in others, have also been reported [77].

One of the core symptoms of autism spectrum disorder is restrictive and repetitive behavior, such as hand flapping, lining up items, or echolalia, which interfere with the individual’s ability to engage in certain activities and have a negative impact on social life. In the treatment of restricted and repetitive behaviors, oxytocin had no significant effect, with most evidence supporting antipsychotic use in such cases [78].

In a more recent meta-analysis, strong evidence linked low oxytocin levels to autism in children. Furthermore, adults with autism had indistinguishable oxytocin levels compared to neurotypical adults, suggesting a possible higher level of oxytocin in neurotypical children that could promote social interaction and development, with a decline in adulthood. Oxytocin treatment in childhood could be a lead candidate in further studies for elevating key social deficits and improving neurological development in children with autism [79].

### 5.2. Oxytocin and Schizophrenia

Oxytocin may be relevant in other types of mental illness, such as schizophrenia. This is not entirely surprising given the social deficits associated with this mental illness. Significant evidence has shown an inverse correlation between plasma oxytocin levels and the severity of schizophrenia symptoms, suggesting that oxytocin may play an important role in the etiology of schizophrenia. Much lower concentrations of oxytocin have been identified in association with more severe symptoms.

Lower plasma oxytocin levels correlate with all symptoms of schizophrenia, but the most consistent evidence has been shown for negative and cognitive symptoms. The significance of these results has not yet been clarified and may have several explanations. A low level of oxytocin could have causal connotations; it could be an effect of the evolution of the disorder or even a response to the antipsychotic treatment [80]. The correlation between the severity of positive symptoms and plasma oxytocin levels has not been consistently supported. Thus, one study found that female patients diagnosed with schizophrenia and positive symptoms also had higher levels of plasma oxytocin compared to male subjects [81]. In contrast to positive symptoms, in the case of negative symptoms, there is consistent evidence of an inverse association between a symptoms’ severity and plasmatic oxytocin levels and between negative symptoms and oxytocin levels in cerebrospinal fluid [82,83]. Moreover, some genetic studies have shown an association between oxytocin levels and negative symptoms. For example, an association between negative symptoms in patients with schizophrenia and a variant of the oxytocin gene (*rs2740204*) has been identified. Studies report a significant association between negative symptom scores as measured by the PANSS scale (positive and negative schizophrenia symptoms scale) and oxytocin receptor variants *rs53576* and *rs237885* [84]. Other studies have shown a significant association between emotional withdrawal typical of schizophrenia and the *rs53576* oxytocin receptor gene variant [85].

Regarding the cognitive component in schizophrenia, it is known that higher peripheral oxytocin levels have often been associated with improved social knowledge and prosocial behavior and better recognition of emotions. Higher plasma oxytocin levels were also associated with avoidant behavior toward faces that expressed anger, better emotion recognition, and more accurately coding socially-relevant information in schizophrenia [86,87]. Furthermore, a higher concentration of oxytocin leads to an improvement in the speed of information processing, as well as an increase in working memory [88]. Thus, intranasal oxytocin as an augmentation to antipsychotic treatment may be a candidate for ameliorating negative and positive symptoms and may help restore social cognitive deficits [89].

However, not all studies have observed these correlations, and a general conclusion cannot be formulated yet. Therefore, the significance of oxytocin in schizophrenia is far from being fully elucidated.

### 5.3. Oxytocin and Personality Disorders

In terms of correlating oxytocin levels with personality, previous studies have shown that more sociable people tend to have higher levels of oxytocin. In the case of borderline personality disorder, they have a high level of stress, hostile and impulsive behaviors, but also major social difficulties. It seems that people with borderline disorders tend to interpret the expressions of ambiguous faces as anger and hostility and show marked and prolonged reactions in response to threatening social cues, associated with more intense and prolonged amygdala responses [90]. Because there is evidence that oxytocin has roles in improving facial recognition and possibly avoiding negative social information, it has been investigated whether patients with borderline personality disorder would benefit from oxytocin administration. Subjects with borderline personality disorder showed greater initial changes in the following signs of social reactivity (faster gaze fixation and increased amygdala activation) in response to angry faces compared to the control group. These abnormal behavioral and neuronal patterns were normalized after exogenous oxytocin administration [91]. Oxytocin could have the effect of reducing hypersensitivity to perceived social threats and thus reduce aggressive and angry behavior in borderline personality disorder.

Antisocial personality disorder includes a key symptom: failure to conform to social norms, thus, oxytocin, with its prosocial effect, could have a potential clinical application for these patients. Oxytocin administration has the effect of correcting deficits in recognizing fearful or happy faces and improves compliance in performing various tasks such as social dilemmas, memory under peer pressure, tasks with monetary involvement, visual search, competitive and non-competitive coin-tossing tasks, prisoners dilemma tasks, and other. It was also observed that compliance and conformity were increased within a group, possibly increasing, at the same time, the vulnerability to peer pressure, posing a problem for institutionalized patients. Overall, oxytocin had beneficial effects on socially positive and non-criminogenic behaviors, with some studies supporting the opposite fact. Probably these discrepancies had a high dependence on social context [92].

### 5.4. Depression and Oxytocin

Studies show that neuropeptides can be mediators in regulating affect and may be a key element in maintaining mood and preventing anxiety. The relationship between neuropeptides and monoamines indicated by biochemical studies could explain the involvement of oxytocin in the pathogenesis of affective disorders. The oxytocin–depression relationship is further supported by clinical and animal studies, with oxytocin having a positive role in depression, and oxytocin imbalances are more common in depression [93].

In postpartum depression, studies have found that oxytocin levels are generally lower compared to control, and some studies suggest that low levels of oxytocin during pregnancy may have a predictive role for postpartum depression [94,95]. However, there are contradictory data on the effects of oxytocin and the affective disposition of pregnant women who needed oxytocin. Oxytocin administration was more frequently associated with postpartum depression. These kinds of results need to consider a multifactorial substrate (hormonal, genetic, and social) that may influence the effects of oxytocin. On the other hand, some pregnant women require perinatal administration of oxytocin, and this may indicate that those women may already have certain hormonal and neuropeptide deficiencies that may predispose them to further development of depression [96]. It has also been observed that early cessation of breastfeeding has been associated with postpartum depression, and the insufficient release of oxytocin may explain this association [97]. A recent study found that oxytocin released during lactation moderates the increase in cortisol levels in response to stress in women with depressive symptoms [98].

Starting from the notion that oxytocin could promote social behavior and a decreased response to stress, various research has tried to study the relevance of oxytocin in depressive disorders, not only in association with the postpartum period. Clinical evidence indicates that the oxytocin system may be unbalanced in major depression. Regarding clinical trials comparing serum oxytocin levels and peripheral cortisol levels in depressed patients, the results were different. Several studies did not identify significant differences between these parameters in depressed patients compared to control subjects [99,100,101], while some identified a decrease in oxytocin levels in patients with depression [102] or even an increase [103,104].

When depression was associated with fibromyalgia, the fall in oxytocin was even more pronounced. Different types of depression may be associated with different levels of oxytocin precisely because of these differences in clinical manifestation. Also, somatoform manifestations are correlated with a higher concentration of oxytocin, even when suffering from depression. At the same time, patients who are more socially withdrawn or who have this symptom as the dominant trait in depression may have a lower level of oxytocin compared to other patients and more so than patients with impulsivity as the main trait. Sex is another very important element in interpreting changes in the peripheral level of oxytocin. Lower levels of oxytocin were found in female patients compared to male subjects, both in the presence of unipolar depression and in the presence of bipolar depression [105].

The mesocorticolimbic pathway, wherein dopamine interacts with oxytocin as a major function, is extremely important in achieving behaviors associated with rewards such as addiction, motivation, survival, diet, and sexual activity [106]. Oxytocin may be involved in changing how the person with depression is interpreting reality in such a way that social interactions may be interpreted as negative or hostile. In consequence, a dysfunction of the oxytocin system could predispose asocial behavior [107]. There are reports in the literature that oxytocin levels change in depression before and after treatment with citalopram. The authors observed significant changes after the appearance of the therapeutic response. The data indicate that oxytocin may play an important role in mediating the clinical effect of SSRI [55]. There is also evidence that the administration of sildenafil, a molecule commonly used in sexual dysfunction, also stimulates the release of oxytocin, thereby having an antidepressant effect [108]. The same antidepressant effect was obtained in treatment with carbetocin, an oxytocin agonist. Administration of carbetocin to experimental animals with a behavioral pattern of depression had antidepressant effects sustained by specific behavioral indices (swimming, immobility time) similar to imipramine, a tricyclic antidepressant [109]. At the same time, clinical trials indicate possible beneficial effects of oxytocin administration in other psychiatric conditions such as anxiety, schizophrenia, autism, drug addiction, or anorexia, as mentioned above in the paper.

#### Oxytocin and Psycho-Somatic Manifestations in Affective Disorders

Somatic manifestations of affective disorders are extremely and commonly associated with mental symptoms. Virtually any organ with vegetative innervation can show changes that reflect emotional disturbances. Thus, we may experience neurological, respiratory, cutaneous, muscular, genitourinary, cardiovascular, or digestive symptoms in depression or anxiety.

Regarding sleep disorders, there is evidence that oxytocin may have hypnotic effects. The link between oxytocin and sleep could be explained by the HPA axis. In one study, the animal subjects were put in stressful conditions and were administered oxytocin and an oxytocin antagonist intracerebroventricularly. This led to a change in the sleep pattern, by decreasing REM sleep and increasing neural activity, with high-frequency waves being recorded on the electroencephalogram. On the other hand, the administration of high doses of oxytocin determined decreased locomotor activity in experimental animals. When oxytocin was administered in relatively low doses, the researchers also observed an anxiolytic effect on animal subjects [110]. The studies report those outcomes dependent on the presence or absence of stress, confirming the connection between the oxytocin system and stress while also suggesting a possible involvement of oxytocin in sleep changes found in depression.

In terms of appetite, weight loss, and weight gain, data from recent studies linking oxytocin to anorexia come to support the notion that oxytocin, most likely stimulated by leptin, could play an anorexigenic role by decreasing food intake, increasing energy consumption, and reducing overall body weight. Leptin, a hormone produced in adipocytes and to a lesser extent by the small intestine, could stimulate certain oxytocin neurons in the hypothalamic paraventricular nucleus, causing a decrease in body weight by inhibiting food intake and increasing energy consumption. Studies show that this effect is mediated through the nucleus of the solitary tract, which supports the idea that oxytocin influences leptin’s action [111]. When oxytocin was administered, decreased food intake was observed, while the administration of an oxytocin antagonist was associated with an increase in food intake [112]. Clinical trials have investigated the relevance of oxytocin in anorexia nervosa, showing different patterns of oxytocin secretion depending on the presence or absence of food stimulus. During fasting, oxytocin levels decrease in anorexic patients, while in the context of dietary intake there is a significant increase in oxytocin levels in patients with anorexia compared to the general population [113].

Genetic studies have also shown that there is nucleotide polymorphism in both the oxytocin receptor gene and the oxytocin gene, which is associated with pathologies that involve hyperphagia, anorexia, and bulimia. Positive correlations have been identified between the G allele of the *rs53576* oxytocin receptor and bulimia. Thus, patients with a G allele had a higher score on the behavioral inhibition system [114]. Differences in some psychological traits have been associated with changes in the oxytocin receptor gene in healthy individuals. The simultaneous presence of anorexia and any of the A alleles for two single nucleotide polymorphisms in the oxytocin receptor gene (*rs53576, rs2254298*) determined an increased severity of the eating disorder, along with an increase in the cognitive distortions and the behaviors associated with both diet and physical appearance. The oxytocin receptor polymorphisms may be a useful endophenotype relevant to anorexia nervosa’s treatment [115].

Sexual function is another significant somatic change in depression. In terms of sexual function, depressed patients report sexual dysfunction at every stage of the sexual response cycle, beginning with arousal, plateau stage, orgasm, and resolution in both men and women [116]. On the other hand, oxytocin is well known to play an important role in reproductive behavior, which includes both the social behavior for creating and maintaining relationships [117]. There are several studies regarding the link between oxytocin and sexual function, with some authors finding a direct involvement of oxytocin in penile erection. Thus, a review published in 2011 claims that oxytocin has a biphasic effect on erection. In other words, at the central level, it has a pro-erectile effect, while at the peripheral level, oxytocin could inhibit the erection. Regarding the next phases of the sexual response cycle, oxytocin is thought to mediate ejaculation, post-ejaculatory detumescence, and the refractory period after orgasm. Possible therapeutic effects targeting the central oxytocin system may be a new target in the development of therapy for the treatment of erectile dysfunction, while intra-cavernous oxytocin injection may be an effective therapy for priapism [118]. However, the role of oxytocin in sexual function, at least in association with depression, is not yet well understood.

Irritable bowel syndrome is an extremely common digestive disorder associated with depression and anxiety. Irritable bowel syndrome is clinically described by recurrent digestive symptoms, with abdominal pain, changes in the frequency and consistency of stool, bloating, digestive tenesmus (the feeling of imperative defecation with incomplete defecation), diarrhea or constipation. Psychosocial stress plays an important role in the etiopathogenesis of irritable bowel syndrome, and patients often report psychological symptoms. These symptoms are either depressive (feeling exhausted, nausea, insomnia, anorexia, low self-esteem, discouragement) or anxious (nervousness, anxiety, obsessive ruminations, and panic attacks). The prevalence of co-association between irritable bowel syndrome and a mental disorder is very high but varies widely between 38% and 100% [119].

The perception of pain is not equal between individuals, even when applying the same stimulus, and the perception of pain is influenced by the psychological state. Therefore, through this strong connection between the brain and the intestine, psychosocial factors, such as emotions, personality traits, and various psychosocial stressors, can influence the response of the digestive system to these factors according to the bio-psycho-social model involving the hypothalamic-pituitary axis through cortisol. There are complex interactions between local intestinal factors (such as inflammation, oxidative stress, and dysbiosis) and the vegetative nervous system. Stress control methods cause improvements in digestive function, and antidepressants may be useful in the therapy of irritable bowel syndrome. The connections between irritable bowel syndrome and psychiatric disorders are extremely valuable because they could facilitate the development of new treatments for irritable bowel syndrome [120].

The connection between oxytocin and cortisol in the irritable bowel predominantly involves endogenous stress-related mechanisms that are activated in a stressful context. Thus, a series of biological reactions are triggered, which ultimately leads to the release into the systemic circulation of cortisol. The oxytocin system modulates the effects of cortisol, partially preventing its negative consequences, such as at the digestive level, where oxytocin could partially block the effects of cortisol. The fact that oxytocin acts on the digestive tract is also evidenced by the presence of oxytocin and its receptors on the entire digestive tract and especially in the intestine. In this context, it has been shown that intestinal myoepithelial cells express oxytocin receptors, protecting against toxins such as inflammation or free radicals [121]. It appears that oxytocin may also be involved in maintaining integrity and homeostasis because, anatomically, the proteins that regulate enterocyte proliferation are in the vicinity of oxytocin receptors. The fact that oxytocin receptors are located at the junction between enterocytes could be an argument in favor of the idea that the function of regulating intestinal permeability may involve oxytocin [122]. These data from the literature offer an important connection between oxytocin, cortisol, and irritable bowel syndrome, suggesting that oxytocin may have a protective role on the digestive system.

## 6. Conclusions

Risk factors for psychiatric disorders are variables that are associated with the increased probability of mental illness. Such risk factors must be present at one life stage and may put the individual at risk of immediate or the later development of a psychiatric disorder. Many of these factors are biological in nature; some are fixed, such as genetic vulnerability, or difficult to influence, like pregnancy and birth complications, brain injuries, or infection, while others are psychosocial and are subject to change. Oxytocin, with its connection to the main neurotransmitter systems, could be a major candidate for mitigating some of the risk factors. The indirect role that oxytocin plays in regulating other neurotransmitters, interpreting different life events, and adapting the individual stress response, could mean that a new line of treatment for preventing the development of psychiatric disorders is accessible for research.

Eating disorders were found to be linked with oxytocin through leptin in the case of anorexia or through changes in oxytocin receptor genes, as seen in cases of bulimia and anorexia, promoting the idea that oxytocin could be used in treatment. Also, the direct connection with the HPA axis with the modulation of cortisol and other cytokines in stress response could bring oxytocin forward as an option for treating irritable bowel syndrome. 

The link with dopamine indicates that oxytocin could be used in a large array of therapies with an impact on addiction, motivation, diet, survival, and sexual activity. Moreover, the connection with the serotoninergic system could have a beneficial impact on antidepressant treatment as an adjuvant or in sustaining symptom remission, or as monotherapy. Thus, oxytocin could become a viable alternative in treating anxiety, depression, drug addiction, or even schizophrenia and autism.

Intranasal administration of oxytocin analogs might have an advantage over other routes when the blood-brain barrier or increasing endogenous oxytocin release is taken into account. The multiple roles that oxytocin has in the psychiatric sphere need to be further investigated in order to establish a solid base for future treatment alternatives. The potential therapeutic uses for oxytocin are of great significance in preventing, managing, and preserving mental health.

## Data Availability

Not applicable.
